# Tracing the Formation of Femtosecond Laser-Induced Periodic Surface Structures (LIPSS) by Implanted Markers

**DOI:** 10.1021/acsami.4c14777

**Published:** 2024-12-27

**Authors:** Robert Wonneberger, Stephan Gräf, Jörn Bonse, Wolfgang Wisniewski, Katharina Freiberg, Martin Hafermann, Carsten Ronning, Frank A. Müller, Andreas Undisz

**Affiliations:** † Institute of Materials Science and Engineering, Chemnitz University of Technology, Erfenschlager Straße 73, Chemnitz 09125, Germany; ‡ Otto Schott Institute of Materials Research, Friedrich Schiller University Jena, Löbdergraben 32, Jena 07743, Germany; § Institute of Solid State Physics, Friedrich Schiller University Jena, Max Wien Platz 1, Jena 07743, Germany; ∥ Bundesanstalt für Materialforschung und -prüfung (BAM), Unter den Eichen 87, Berlin 12205, Germany

**Keywords:** laser-induced periodic surface structures (LIPSS), ion implantation, transmission electron microscopy (TEM), stainless steel, femtosecond laser processing

## Abstract

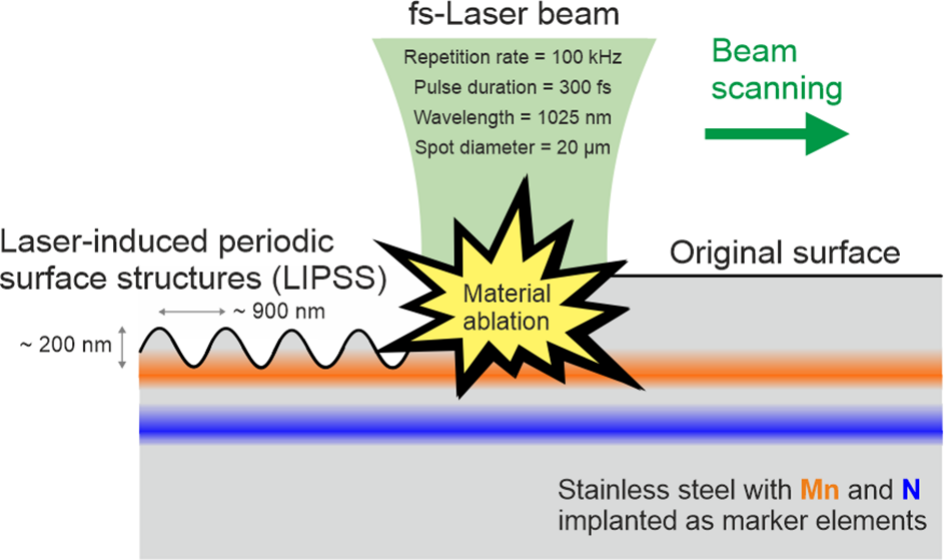

The generation of laser-induced periodic surface structures (LIPSS) using femtosecond lasers facilitates the engineering of material surfaces with tailored functional properties. Numerous aspects of their complex formation process are still under debate, despite intensive theoretical and experimental research in recent decades. This particularly concerns the challenge of verifying approaches based on electromagnetic effects or hydrodynamic processes by experiment. In the present study, a marker experiment is designed to conclude on the formation of LIPSS. Well-defined concentration depth profiles of ^55^Mn^+^- and ^14^N^+^-ions were generated below the polished surface of a cast Mn- and Si-free stainless steel AISI 316L using ion implantation. Before and after LIPSS generation, marker concentration depth profiles and the sample microstructure were evaluated by using transmission electron microscopy techniques. It is shown that LIPSS predominantly formed by material removal through locally varying ablation. Local melting and resolidification with the redistribution of the material occurred to a lesser extent. The experimental design gives quantitative access to the modulation depth with a nanometer resolution and is a promising approach for broader studies of the interactions of laser beams and material surfaces. Tracing LIPSS formation enables to unambiguously identify governing aspects, consequently guiding the path to improved processing regarding reproducibility, periodicity, and alignment.

## 1 Introduction

Femtosecond (fs) laser processing is an attractive method for modifying material surfaces from the micro- to the nanometer range.^1^ Particular interest lies in a specific type of nanostructures, the so-called laser-induced periodic surface structures (LIPSS, ripples). Numerous promising applications of these grating-like LIPSS have recently been demonstrated in the field of surface functionalization.^2^ Examples include structural colors, e.g., for optical effects or safety features, friction and wear reduction, sensors, modification of the surface wetting behavior, and antibacterial or cell growth-altering properties for medical implant materials.^3−7^ Despite several decades of research, key aspects regarding the formation of LIPSS remain unclear and are still subject to controversial debates.^8^ The existing approaches to explain the LIPSS formation focus either (i) on electromagnetic effects causing mainly material removal by ablation or (ii) on hydrodynamic processes suggesting the redistribution of material involving melting, melt-displacement, and resolidification.^9^ Assessing which aspects of the proposed approaches (i) or (ii) are actually applicable is of major interest to understand and further develop the technique.^9^ Therefore, one main challenge is to reveal which processes occur in the interaction volume of the laser beam with the material during the few ns of LIPSS generation.^10^ This includes open aspects of, e.g., the amount of material removal, whether LIPSS formation is dominated by ablation, melting and hydrodynamic transport, or microstructural changes that occur in the so-called heat-affected zone (HAZ) of less than a few μm.^11^ These aspects are challenging to examine due to the short time scales and small dimensions,^12^ hindering simultaneous visualization. Access could be gained using suitable markers that do not influence the LIPSS generation.

In this work, ion implantation is used to add low concentrations of marker elements with a well-defined concentration depth profile below the sample surface at a specific depth, an approach, recently used to implant ^55^Mn^+^-ions in stainless steel.^13^ Correlating the concentration profiles of the markers before and after fs-laser irradiation enables to assess the material removal during LIPSS formation. The analysis of cross-sections using transmission electron microscopy (TEM) and energy dispersive X-ray spectroscopy (EDXS) facilitates a nanometer resolution, e.g., to locally determine the modulation depth. Moreover, the analysis of concentration profiles and a potential alteration of the material microstructure enables to draw conclusions regarding the formation of LIPSS, especially whether local melting and resolidification of materials occurred, as recently shown for a NiTi alloy.^14^

## 2 Materials and Methods

### 2.1 Alloy Casting and Surface Preparation

A Mn- and Si-free austenitic stainless steel was prepared, resembling the composition of AISI 316L. The alloy was melted using a cold wall vacuum levitation furnace and the elements Fe, Cr, Ni, and Mo with a purity of at least 99.95%. The final composition, as measured by glow-discharge optical emission spectroscopy (GD-OES), is listed in Table 1. Subsequently, the as-cast sample with a mass of 40 g was cold rolled and recrystallized at 1050 °C in consecutive steps to obtain sheets with a final thickness of ∼1 mm. The recrystallized steel sheet was cut to rectangular segments of ∼5 mm^2^ × 1 mm in size using a CO_2_ laser. The sample surface was ground using SiC paper with decreasing grain sizes and then polished to a mirror-like finish with diamond suspensions down to particle sizes of 1 μm. All samples were cleaned with ethanol in an ultrasonic bath and dried by using pressurized air.

**Table 1:** Average Composition (wt %) with the Standard Deviation of Six GD-OES Measurements of the As-Cast Alloy

Fe	Cr	Ni	Mo	Mn	Si
balance to 100%	19.7 ± 0.3	11.2 ± 0.2	2.8 ± 0.1	0.0	0.0

### 2.2 ^55^Mn^+^- and ^14^N^+^-Ion Implantation into the Sample Surface via Ion Irradiation

^55^Mn^+^- and ^14^N^+^-ions were subsequently implanted into the polished sample with an ion energy of 380 keV. The conditions for ion implantation of each marker element were chosen carefully so that the ion ranges are in the range of the documented laser modulation depth for steels during the subsequent laser treatment for LIPSS formation.^15^ The typical Gaussian-shape concentration depth profile of each implanted marker was simulated using the software package stopping and range of ions in matter (SRIM)^16^. The ion ranges were calculated to 135 and 340 nm for the ^55^Mn^+^- and ^14^N^+^-ions, respectively. Details of the implantation parameters and simulation results are given in Table 2.

**Table 2:** Ion Irradiation Parameters for Mn und N into Austenitic Stainless Steel AISI 316L^a^1

element	ion energy (keV)	ion fluence (10^16^ cm^–2^)	incidence angle (deg)	peak concentration (wt %)	ion range (nm)	straggling (nm)
^55^Mn^+^	380	7.3	7	5.9	135	55
^14^N^+^	380	4.9	7	0.8	340	82

a The ion range, straggling, and peak concentration was simulated with the software package SRIM using a mass density of 7.99 g/cm^3^ and the alloy composition given in Table 1.

### 2.3 Femtosecond Laser Processing

A commercial fs-laser system (JenLasD2.fs-laser, Jenoptik AG, Germany) with a wavelength of λ = 1025 nm, a pulse duration of τ = 300 fs, and a peak fluence of *F*_0_ = 1.5 J/cm^2^ was used to form the LIPSS. The pulse repetition rate was *f*_rep_ = 100 kHz, which corresponds to a temporal pulse separation of 10 μs. The LIPSS (type I low-spatial frequency LIPSS, LSFL-I) were generated by scanning the focused Gaussian laser beam with a focal 1/*e*^2^-spot diameter of 2*w*_f_ = 20 μm unidirectionally across the sample surface at ambient air with a scanning velocity of *v*_*x*_ = 0.67 m/s and a line hatch distance of Δ*y* = 6 μm. This led to effective pulse numbers per beam spot diameter of ∼3 (1D) and ∼8 (2D), accordingly.^9^ After LIPSS generation, the sample was cleaned in an ultrasonic bath with acetone and isopropanol for ∼10 min.

### 2.4 Analysis of Elemental Distribution and Microstructural Changes Using TEM

TEM lamellae were prepared in cross-section using focused ion beam milling in a Helios NanoLab 600i (FEI) dual beam scanning electron microscope (SEM) as previously described.^17^ The sample surface was covered with protective Pt using first electron (e-Pt) and then ion (i-Pt) beam deposition from a precursor gas. Lamellae were subsequently cut with the Ga-ion beam, operating at 30 kV and a current of 2.5 nA. The transfer to Cu-grids was performed using an OmniProbe micromanipulator stage system. Subsequently, a final thinning procedure was applied, during which the acceleration voltage and ion beam current were reduced, respectively, in four consecutive steps down to 5 kV/15 pA with stage tilts of 50.5–53.5°. SEM imaging was performed at 5 kV by using a secondary electron (SE) detector.

**Figure 1:**
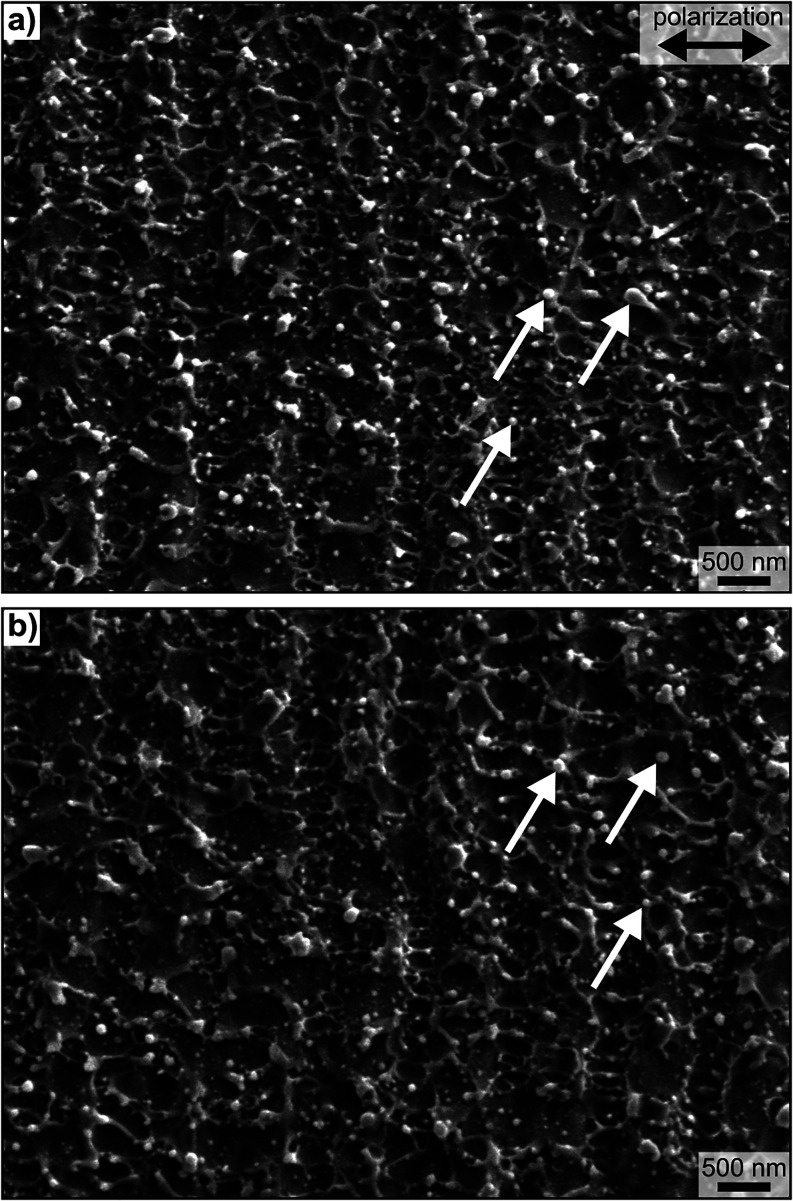
SEM micrographs providing an overview of (a) a polished sample as well as (b) a polished and ^55^Mn^+^- and ^14^N^+^-ion implanted sample after LIPSS generation. Some spherical heterogeneities, exemplarily marked by the white arrows, are randomly distributed over the surface. The horizontal polarization direction of the laser beam for both samples is depicted in (a).

The scanning TEM-(STEM-) mode was utilized to acquire STEM-EDXS maps using a C_s_-probe corrected JEOL NEOARM F200 equipped with two JEOL 100 mm^2^ SSD detectors. The STEM-EDXS spectra were subjected to a background subtraction by postprocessing with the software package Pathfinder 2.6 (Thermo Fisher Scientific) using the Filter-Fit method for fitting. For noise reduction, the total counts for each pixel were enhanced by 3 × 3 binning, summing up the counts of the eight adjacent pixels. High-resolution TEM (HR-TEM) and STEM imaging were performed by using a bright field (BF) detector.

## 3 Results and Discussion

Figure 1 presents top-view SEM micrographs of LIPSS generated on polished samples of the untreated and implanted stainless steel for comparison. As it is typical for metals, LSFL with spatial periods Λ in the order of the laser wavelength λ (Λ ∼ 900 nm) and an orientation perpendicular to the fs-laser beam polarization, indicated in Figure 1a, are discernible. The morphology of the LSFL on both sample conditions is similar, implying that the ion implantation had no significant effect on the LIPSS formation.

This characteristic topography, as well as the formation of spherical heterogeneities (white arrows), often denoted as nanoparticles, are also similar to previous work concerning LIPSS formation on commercially available AISI 316L containing small amounts of Mn and Si.^15,18^ Accordingly, LIPSS formation is insensitive to minor concentrations of accompanying or implanted elements and possible concomitant changes to the microstructure.

**Figure 2:**
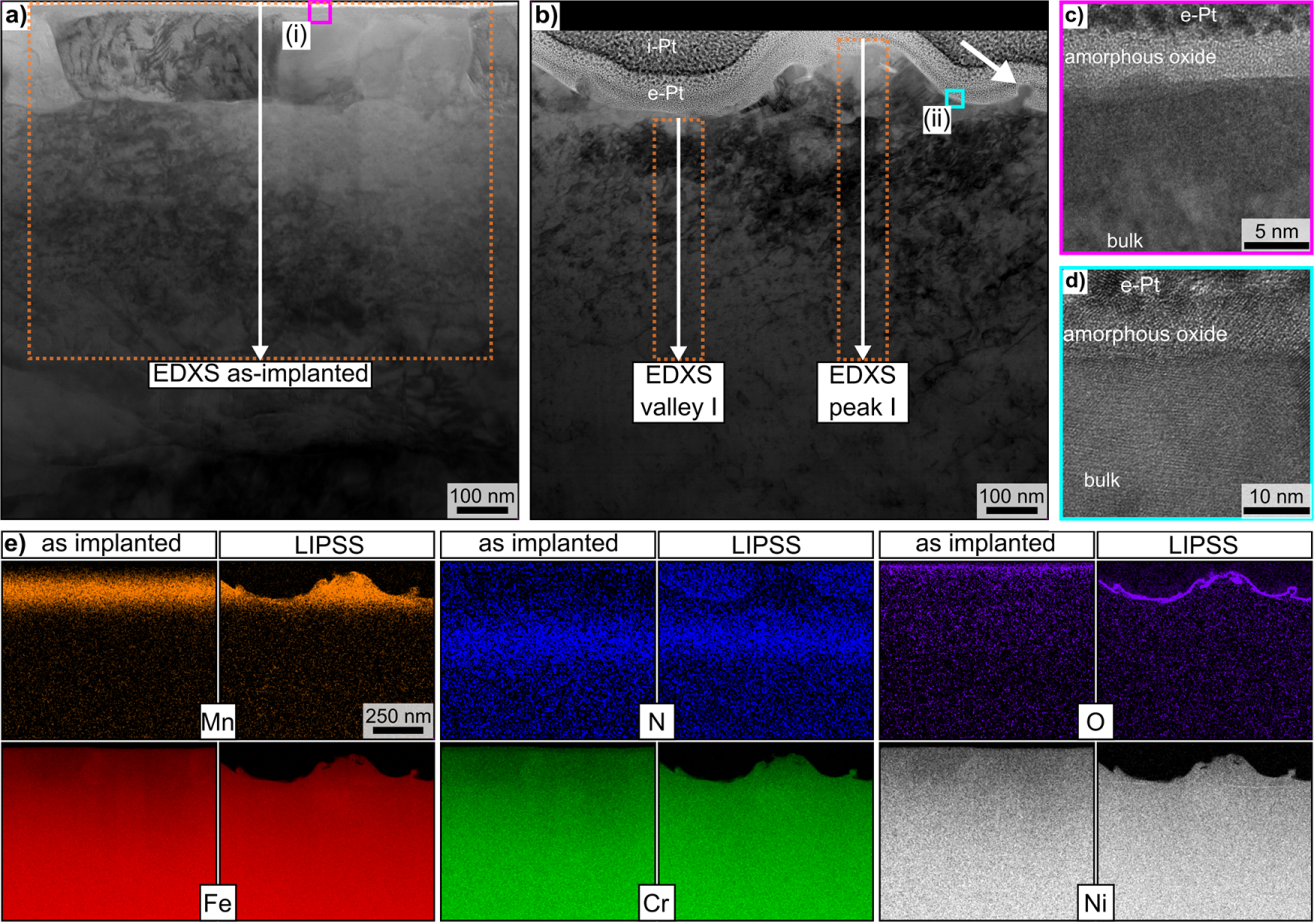
STEM-BF micrographs of a cross-section prepared perpendicular to the surface of the (a) cast and polished stainless steel after the implantation of ^55^Mn^+^- and ^14^N^+^-ions and (b) after LIPSS formation. The micrographs are aligned with each other according to the concentration profiles of the implanted markers Mn and N to provide visual access to the relative positions of the surface before and after LIPSS generation. The short white arrow highlights heterogeneity on the surface. The area of frame (i) is presented in (c) and of frame (ii) in (d), visualizing an amorphous oxide layer on both samples. STEM-EDXS maps of the respectively stated elements in (e) were acquired from the cross-sections featured in (a) and (b). Data of these EDXS maps are accumulated to quantitative EDXS-profiles along the white arrows in (a) and (b) that are presented and further discussed below.

Figure 2a presents a cross-section of the as-implanted sample without fs-laser irradiation. As assumed, there are minor changes to the microstructure near the surface of the sample in the form of additional grain boundaries due to the ion implantation process. The altered microstructure is limited to the damage range of the implanted ^55^Mn^+^, consistent with the different energies induced during ^55^Mn^+^- and ^14^N^+^-ion implantation. A comparable micrograph of a cross-section prepared after generating LIPSS on the implanted material is presented in Figure 2b. The regular modulation of the LIPSS topography is evident in the alternating peaks and valleys. In both cases, an amorphous oxide layer is visible by its brighter contrast, discernible in Figure 2c,d. The oxide layer on the LIPSS processed sample is ∼10 nm thin, approximately twice as thick as on the as-implanted sample. It is important to note that such microstructural changes in the upper few nanometers of the alloy have a subordinate influence on the generation of LIPSS, as their typical modulation depth is about 200 nm on the stainless steels AISI 304 and AISI 316L, respectively.^15,19^

The STEM-EDXS maps presented in Figure 2e show the comparison of implanted Mn and N markers parallel to the original surface. Whereas the maximum of the Mn concentration profile is within the interaction volume of the laser beam, the maximum of the N concentration profile is well below it and thus is a suitable reference to determine the distance to the original surface. The measurements show some intensity of the N signal in the region of deposited Pt. This is most likely an artifact of the lamella preparation and material removal using FIB milling. The protective coating consists of Pt nanoparticles which are known to adsorb N under appropriate conditions.^20^ The mild but gradually decreasing signals of Fe, Cr, and Ni measured for the as-implanted sample near the surface indicate their relative depletion in this area, which is caused by the added amount of Mn. However, these main constituents of the steel alloy remain homogeneously distributed throughout the samples after fs-laser processing.

**Figure 3:**
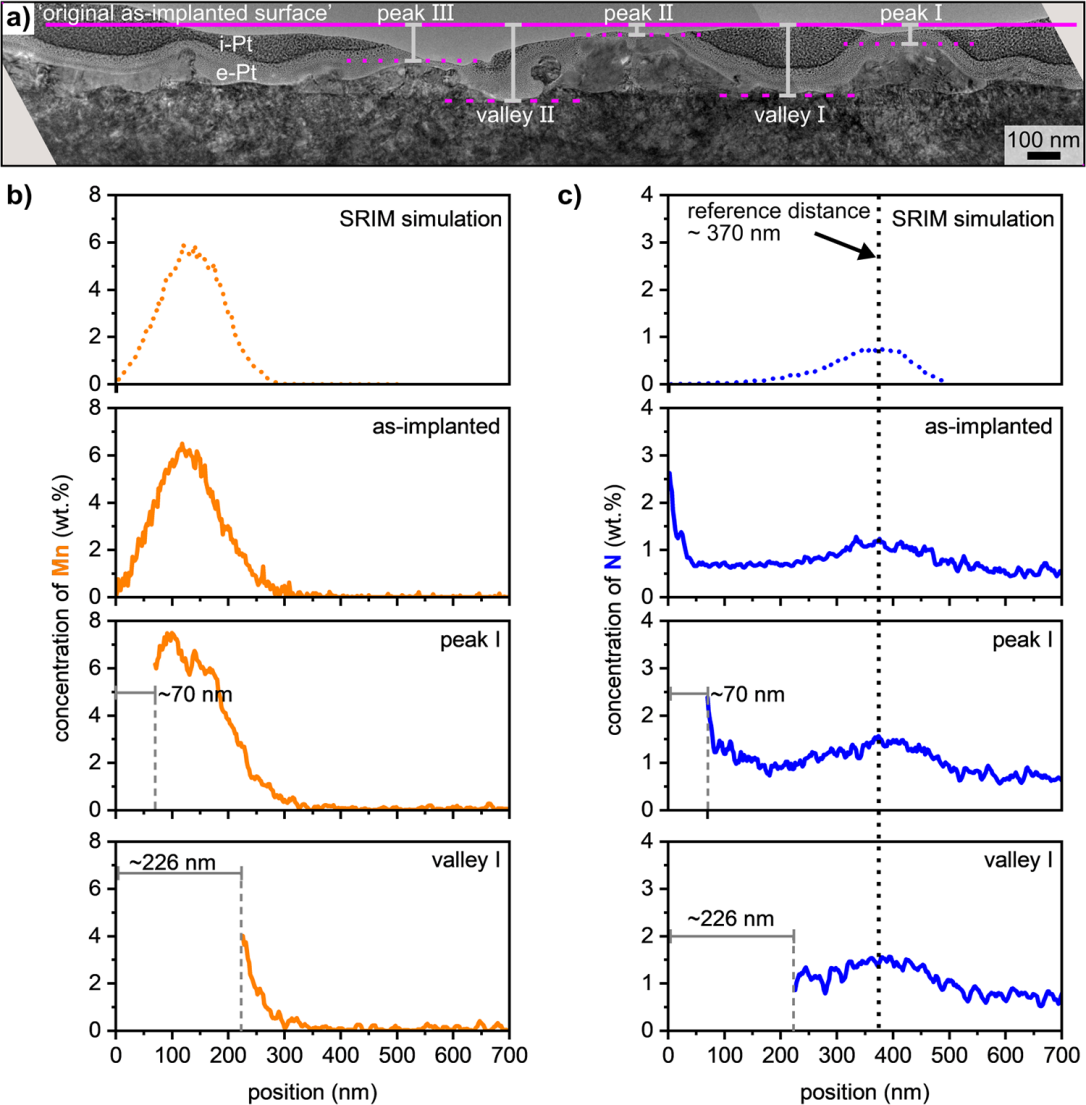
Low magnification TEM-BF micrograph of the cross-section featured in Figure 2b. Peaks (numbered I, II, and III) and valleys (numbered I and II) are marked in (a). STEM-EDX line scans acquired from the regions marked in Figure 2a,b (“EDXS as-implanted,” EDXS valley I, and EDXS peak I) are shown in (b) for Mn, in (c) for N and are respectively compared with the corresponding SRIM simulation. The original “as-implanted” surface is marked by a solid horizontal magenta line in (a) as a reference.

Figure 3a presents an overview of the sample featured in Figure 2b, where the positions “valley I” and “peak I” are the same positions of the profiles “EDXS valley I” and “EDXS peak I.”

The concentration profiles of N and Mn along these line scans are, respectively, compared to that of “EDXS as implanted” in Figure 2a. Please note that these three profiles were aligned to the N maximum, marked by the dotted line in Figure 3c. Hence, the similar slope in the Mn-profile indicates that this element is not redistributed during LIPSS formation. As the N maximum was initially at the same depth in both samples, the difference between the distances from the surfaces to the N maximum between the as-implanted sample and the LIPSS sample represents the material removal at each local position. For these cross-sections, it was determined to be ∼70 nm at the position of peak I and ∼226 nm for the position of valley I. Consequently, the resulting modulation depth (peak-valley height) at this location is ∼156 nm, which is close to the documented modulation depth of LIPSS on stainless steel mentioned above. Manually superimposing these distances onto the micrograph in Figure 3a enables to define the position of the original as-implanted surface which is represented by the horizontal solid magenta line. Correlating it to the further peak and valley positions in the TEM-BF micrograph shows that the material removal at the LIPSS peaks ranges from 42 to 120 nm, whereas it ranges from 226 to 242 nm in the LIPSS valleys.

**Figure 4:**
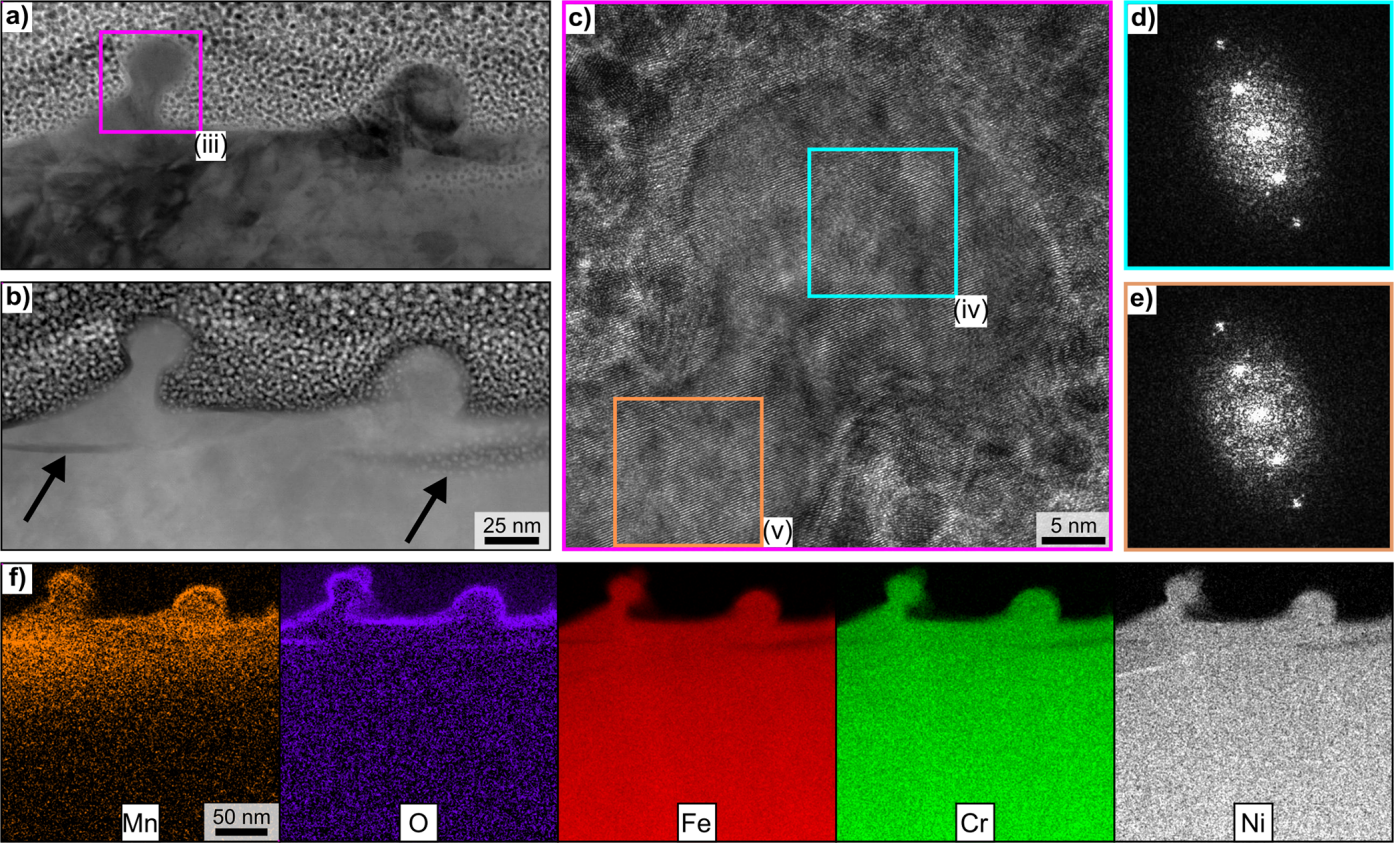
STEM micrographs showing (a) a bright field and (b) a high-angle annular dark field image of two heterogeneities on the surface. An HR-TEM micrograph of the area in frame (iii) is shown in greater detail in (c). The sample was tilted 7° along the *x*- and *y*-axes in a low-oriented zone axis for imaging. 2D-FFT patterns acquired from the areas in frames (iv) and (v) are shown in (d) and (e). EDXS element maps of the whole area are presented in (f).

The presented experiment enables to measure the material removal above the topographic peaks, proving that the LSFL-I type LIPSS studied here are formed in the ablative laser processing range, which is in agreement with the approach explaining LIPSS formation based on electromagnetic effects.^9^ It confirms the assumption of selective ablation within the focal spot resulting from the interference of the incident laser radiation with excited electromagnetic surface waves including surface plasmon polaritons (SPP). A more detailed discussion of the presented results in the context of the approaches available to explain LIPSS formation requires taking microstructural features into account, e.g., the observed heterogeneities marked by white arrows in Figure 1 and presented in detail in Figure 4a–c. Such spherical heterogeneities at the surface are characteristic for LIPSS and often denoted as “nanoparticles.”^21,22^ However, it has been reported that even an ultrasonic bath does not remove them completely.^14^ The TEM micrographs show that at least some of the protruding heterogeneities are essentially part of the alloy. This is proven by the coherent lattice planes of the entire heterogeneity matching the lattice of the adjacent bulk, visualized by the 2-dimensional fast Fourier transform (2D-FFT) images in Figure 4d,e, acquired in the bulk and particle interior, as marked by squares in Figure 4c. It is likely that such features may affect or even enhance the formation of LIPSS via their characteristic electromagnetic scattering behavior for subsequent laser pulses.

Furthermore, subsurface gaps of width of several nanometers are oriented roughly parallel to the original surface and marked by arrows in Figure 4b. The ion implantation process itself is unlikely to be the cause, as such gaps were not observed in the as-implanted sample.

Regarding the LIPSS formation process, these results indicate that, in addition to an ablative process, the fs-laser irradiation melted the alloy and led to protuberances in the melt. Not all of them fully detach to form independent particles driven by the melt surface tension. Instead, because of the high cooling rate, the protuberances solidify together with the molten alloy. This scenario is supported by the absence of an oxide layer between the heterogeneity and the bulk, discernible in the EDXS map in Figure 4f. The other EDXS element maps confirm the homogeneous distribution of the alloy components as well as-implanted Mn throughout both heterogeneities. The formation of such connected heterogeneities is in agreement with advanced simulations of Shugaev et al. numerically coupling a two-temperature model (TTM) with molecular dynamics (MD) simulations after fs-laser excitation of a metallic Cr film.^10^ However, it should be noted that these MD-TTM simulations calculated the response of the material to a single ultraviolet fs-laser pulse, whereas several laser pulses contribute to LIPSS formation in the present study. The morphology and composition of peak I in Figures 2 and 3 enable to address some open questions concerning whether electromagnetic effects or hydrodynamic processes play a major role. The comparable slope of the Mn concentration profile before and after LIPSS indicates that the peak was formed neither from the melt nor from redeposited or redistributed material. Otherwise, the initial Gaussian concentration profile of Mn would have to change to a homogeneously broadened distribution of Mn due to melt formation or to exhibit a maximum Mn concentration at the surface resulting from deposited Mn enriched alloy. Additionally, the Mn-maximum of peak I is below the surface or, in other words, the Mn concentration decreases towards the surface. This would not be in agreement with a dominant role of hydrodynamic processes,^23^ where melt with an increased Mn concentration from the valleys would get pushed towards the peaks. Finally, a sole material reorganization and redistribution of the material excited by the laser as described in the scenario of self-organization^24,25^ can be excluded here because, without ablation, the peaks in Figure 3a then would have to protrude beyond the original surface. Therefore, our results are most consistent with the approach focusing on electromagnetic effects, where LIPSS formation is dominated by material removal due to ablation.

## 4 Conclusion

The implantation of ^55^Mn^+^- and ^14^N^+^-ions is suitable to generate defined concentration depth profiles, serving as markers in a cast Mn- and Si-free stainless steel AISI 316L. The formation of LSFL-I type LIPSS is unaffected by minor concentrations of accompanying or implanted elements and possible concomitant changes to the microstructures because the generated LIPSS observed in the studies are similar to LIPSS presented in the literature for commercially available AISI 316L. The material removal and LIPSS modulation depth were quantified using TEM techniques, which enable to compare and evaluate the implanted concentration depth profiles before and after LIPSS generation. Taking microstructural changes into account enables to propose that the LIPSS formation is dominated by near-laser-wavelength-sized and polarization-ruled material removal due to ablation rather than a hydrodynamic transport of the melt with redistribution of material at and to above the initial surface plane. The morphology of the observed spherical surface heterogeneities is, like the often denoted and characteristic nanoparticles, formed during LIPSS generation. The heterogeneities are probably the result of rapidly solidified protuberances formed in the melt during LIPSS formation, indicating the occurrence of hydrodynamic effects on the few-nanometer-scale (subwavelength).
